# MR-CBCT image-guided system for radiotherapy of orthotopic rat prostate tumors

**DOI:** 10.1371/journal.pone.0198065

**Published:** 2018-05-30

**Authors:** Tsuicheng D. Chiu, Tatsuya J. Arai, James Campbell III, Steve B. Jiang, Ralph P. Mason, Strahinja Stojadinovic

**Affiliations:** 1 Department of Radiation Oncology, University of Texas Southwestern Medical Center, Dallas, Texas, United States of America; 2 Department of Radiology, University of Texas Southwestern Medical Center, Dallas, Texas, United States of America; National Cheng Kung University, TAIWAN

## Abstract

Multi-modality image-guided radiotherapy is the standard of care in contemporary cancer management; however, it is not common in preclinical settings due to both hardware and software limitations. Soft tissue lesions, such as orthotopic prostate tumors, are difficult to identify using cone beam computed tomography (CBCT) imaging alone. In this study, we characterized a research magnetic resonance (MR) scanner for preclinical studies and created a protocol for combined MR-CBCT image-guided small animal radiotherapy. Two in-house dual-modality, MR and CBCT compatible, phantoms were designed and manufactured using 3D printing technology. The phantoms were used for quality assurance tests and to facilitate end-to-end testing for combined preclinical MR and CBCT based treatment planning. MR and CBCT images of the phantoms were acquired utilizing a Varian 4.7 T scanner and XRad-225Cx irradiator, respectively. The geometry distortion was assessed by comparing MR images to phantom blueprints and CBCT. The corrected MR scans were co-registered with CBCT and subsequently used for treatment planning. The fidelity of 3D printed phantoms compared to the blueprint design yielded favorable agreement as verified with the CBCT measurements. The geometric distortion, which varied between -5% and 11% throughout the scanning volume, was substantially reduced to within 0.4% after correction. The distortion free MR images were co-registered with the corresponding CBCT images and imported into a commercial treatment planning software SmART Plan. The planning target volume (PTV) was on average 19% smaller when contoured on the corrected MR-CBCT images relative to raw images without distortion correction. An MR-CBCT based preclinical workflow was successfully designed and implemented for small animal radiotherapy. Combined MR-CBCT image-guided radiotherapy for preclinical research potentially delivers enhanced relevance to human radiotherapy for various disease sites. This novel protocol is wide-ranging and not limited to the orthotopic prostate tumor study presented in the study.

## Introduction

Modern preclinical irradiation platforms enable sophisticated image-guided radiotherapy (IGRT) studies. Typically, the IGRT is enabled by utilizing volumetric cone beam computed tomography (CBCT) with excellent bone visibility, but a major challenge is the level of soft tissue contrast intrinsic to CBCT. Cancerous lesions may be difficult to identify when surrounded by soft tissue, *e*.*g*., orthotopic prostate cancer. To overcome the analogous challenge in human radiation therapy, patients are usually scanned with MR in addition to CT simulation. The treatment plans are then based on fused MR-CT images [1–4]. Currently, such hardware and software solutions are generally not available for preclinical studies or they are rare and in development stage [5–7]. It may take numerous years to gather preliminary data from preclinical studies regarding the efficacy, toxicity, and safety essential to assure a smooth translation from bench to bedside. Two key components for a successful transition are dosimetric accuracy [8] and the ability to mimic the clinical setting and workflow in preclinical studies.

In the past decade, several small animal radiotherapy platforms have been developed [7, 9–15] leading to sophisticated commercially available products. Notably, Precision X-Ray Inc. (PXI, North Branford, CT, USA) developed the X-Rad series [4] and Xstrahl Life Sciences introduced the small animal radiation research platform (SARRP) [16, 17] and now offer assorted options for IGRT for small animal preclinical studies [9, 18–25]. One option is an integrated treatment planning system (TPS): SmART-Plan [18] was developed for the PXI units and Muriplan for XStrahl. Both SmART-Plan and Muriplan use graphics processing unit (GPU) architecture for Monte Carlo and superposition/convolution based accelerated 3D dose computation. However, neither offers integrated MR image-guided radiation therapy functionality to overcome low CT contrast.

CBCT generally fails to provide adequate tissue contrast to reliably define the location and treatment volume of orthotopic prostate tumors. Since MR imaging is often used to monitor tumor growth in rats, it appeared attractive to use the well-defined soft tissue anatomy to guide radiotherapy [26]. We combined MR data with the corresponding CBCT data to enable MR-CBCT image-guided SmART planning for radiotherapy of orthotopic prostate tumors. Two MR-CBCT compatible geometry phantoms were developed for quality assurance (QA) tests and to evaluate the end-to-end system performance and accuracy. In addition, a dual modality MR-CBCT based preclinical workflow was designed and implemented for small animal radiation therapy. This novel protocol enables added functionality for XRAD 225Cx SmART-Plan, so that the structures can be delineated on fused MR-CBCT images while dose calculation is based on CBCT images. An MR-CBCT based preclinical workflow was commissioned and implemented for small animal radiotherapy.

## Materials and methods

### MR-CBCT calibration and geometry phantoms

**Fig 1:**
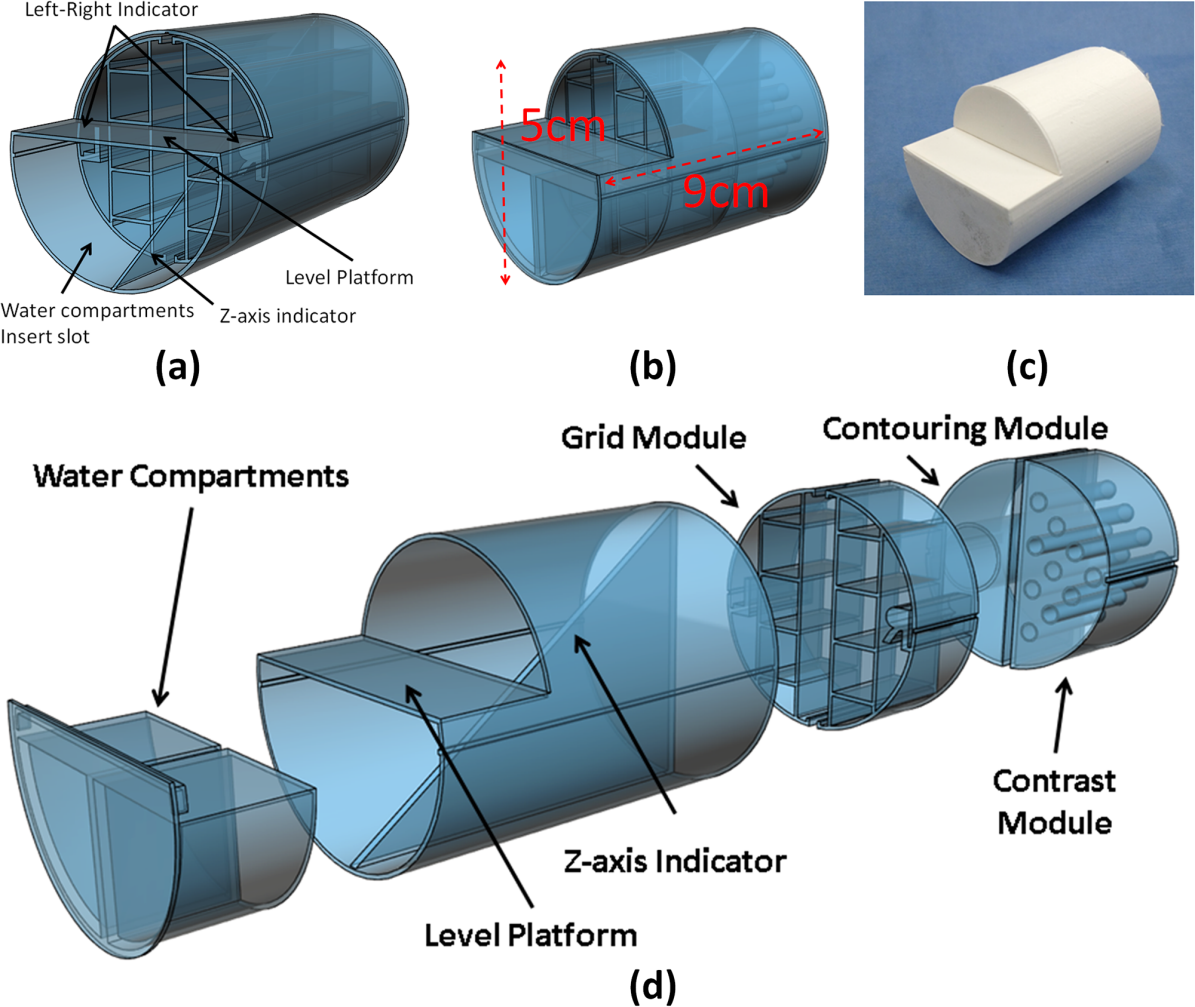
Two preclinical MR-CBCT imaging phantoms. (a) Calibration phantom design (b) Geometry phantom design (c) 3D printed geometry phantom (d) Geometry phantom assembly. The cylindrical phantom was designed to fit within imaging and treatment cradles. The fillable compartments allow inclusion of materials such as water, air, silicone and glass to generate contrast.

Two in-house dual-modality phantoms, MR and CBCT compatible, were designed and manufactured using three-dimensional (3D) printing technology MakerBot Z18 (MakerBot Industries LLC, Brooklyn, NY, USA), as shown in Fig 1, to evaluate MRI using Varian 4.7 T scanner (Agilent Technologies, Santa Clara, CA, USA) and CBCT using XRad-225Cx irradiator (PXI, North Branford, CT, USA). The calibration phantom, Fig 1(a), was used to characterize the geometric distortion of the MR scanner [27]. The geometry phantom, Fig 1(b) and 1(c), was used to facilitate end-to-end testing for combined preclinical MR and CBCT based treatment planning. The internal structures of the geometry phantom, Fig 1(d), were used to compute a coordinate transformation between two imaging datasets.

The calibration phantom, Fig 1(a), is a 9 cm long cylinder with a 5 cm diameter base which can fit in the custom-built animal cradle (Section II.2). It contains six 10 mm × 10 mm × 70 mm rectangular parallelepiped grids with 0.8 mm thick walls spanning the entire imaging field. Furthermore, there are three additional features: the z-axis indicator, the level platform, and the water compartment for MR calibration. The z-axis indicator serves as a reference measure of length along z-axis. The z-axis indicator is also a quality assurance test tool for MR and CBCT image registration check in the axial planes. The level platform has two water compartments intended as a source of water signal for MR scanner calibration. The plateau is utilized for placing a “bull’s eye” level to minimize the rotational setup errors between MR and CBCT scans. The phantom is intentionally designed to be chiral, *i*.*e*., it is distinguishable from its mirror image by introducing the left and right side indicators and the z-axis marks, as illustrated in Fig 1(a).

The phantom was filled with platinum-catalyzed Ecoflex^®^ 00–30 silicone (Smooth-on Inc., Macungie, PA, USA). Phantom MR and CBCT imaging were performed with identical orientation. The fidelity of both imaging modalities was compared to a blueprint design of the phantom. MR images were aligned to CBCT images along z-axis based on the z-axis marks. Then, the spatial correction factors as a function of slice location along the z-axis were obtained by comparing the MR images with the corresponding CBCT images.

The geometry phantom is a 7 cm long cylinder with 5 cm diameter, see Fig 1(d) with the same features as calibration phantom such as water compartments, level platform and z-axis indicator. The geometry phantom has three modules: a grid module, a contrast module, and a contouring module. The grid module was designed for geometric validation subsequent to applied distortion corrections. The module is separated into left and right parts with three grid patterns in each section. Each grid is 10 mm × 10 mm × 20 mm contained by 0.8 mm thick walls. The compartments are filled with three materials: air, Dragon Skin^®^ 20 and Ecoflex^®^ 00–30 silicones. The contrast module is designed to provide intensity variations from different materials for both MR and CBCT scans. Air, water, two silicone compounds, and glass rods are used in the module to provide intensity variability.

The contouring module is used to verify the volume agreement between planning target volume (PTV) in SmART-Plan and the physical target. A glass marble with 15.8 mm diameter is embedded in the module to achieve this purpose.

### MR-CBCT compatible simulation cradle

**Fig 2:**
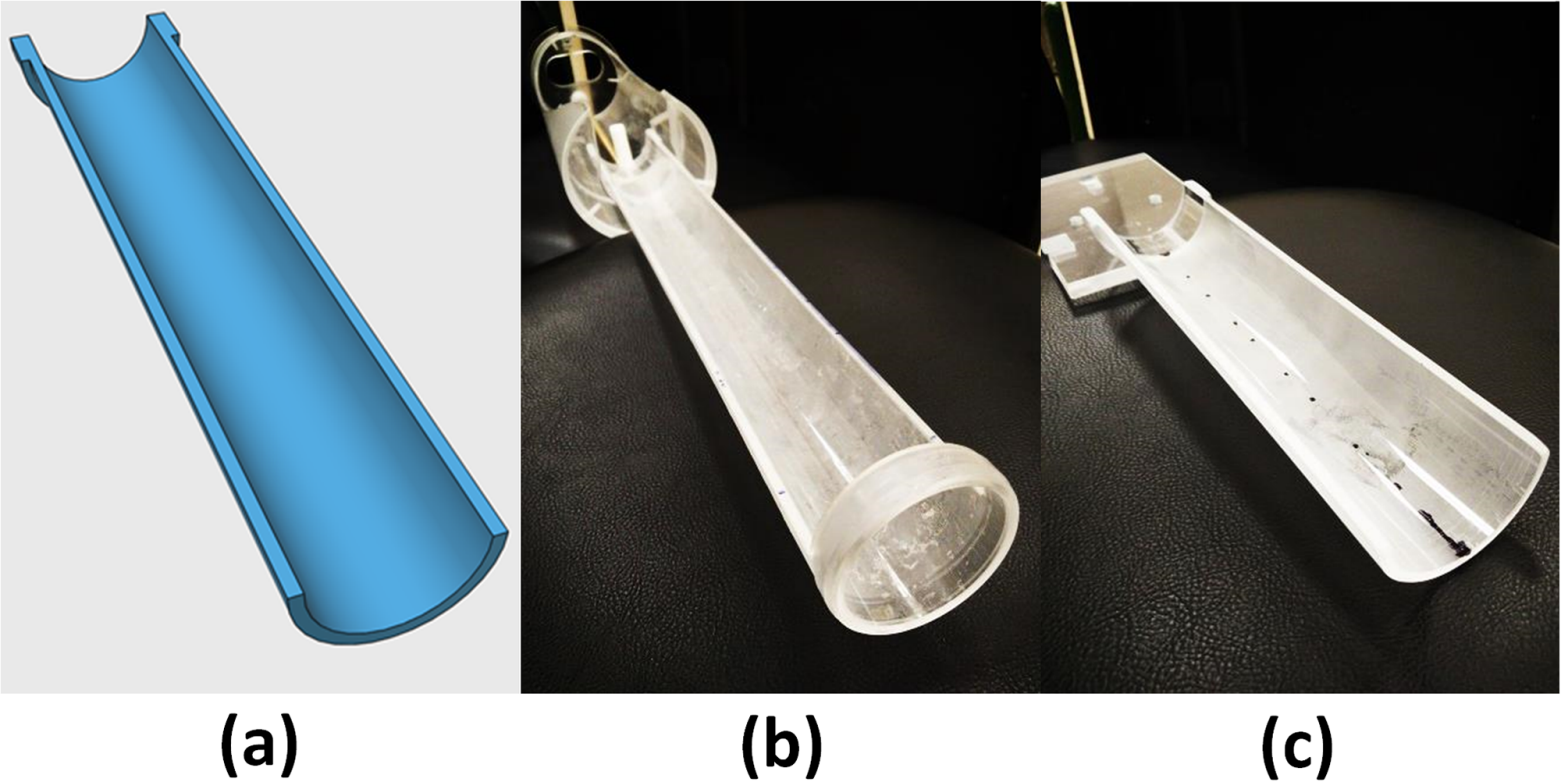
In-house designed MR-CBCT compatible cradle. (a) computer-aided cradle design, (b) Preclinical MR scanning cradle, (c) PXI 225Cx CBCT simulation cradle.

Animal immobilization, ensuring high positioning reproducibility in the MR scanner as well as for on-board XRad 225Cx CBCT imaging, was accomplished using custom-designed MR-CBCT compatible cradles. Fig 2(a) illustrates the computer-aided cradle design essential for the reproducible setup. Two semi-cylindrical cradles with identical curvature were made from acrylic material. The only difference is the docking mechanism used to attach the cradles to the corresponding MR scanner and irradiator hardware. Fig 2(b) shows the MR version with extending apparatus fixed using MR-safe brass screws suitable for the MR coil assembly. Fig 2(c) shows the XRad 225Cx cradle counterpart with identical curvature, but with an attachment compatible to the irradiator’s motorized platform. The rat was imaged and treated in a head-first-supine (HFS) setup. The advantage of curved cradle design is that the most likely animal positioning uncertainty is either translation in superior-inferior (S-I) direction or rotation around S-I axis. Both setup errors can be accounted for by the XRad 225Cx co-registration algorithm.

### Image acquisition

#### MR acquisition

**Fig 3:**
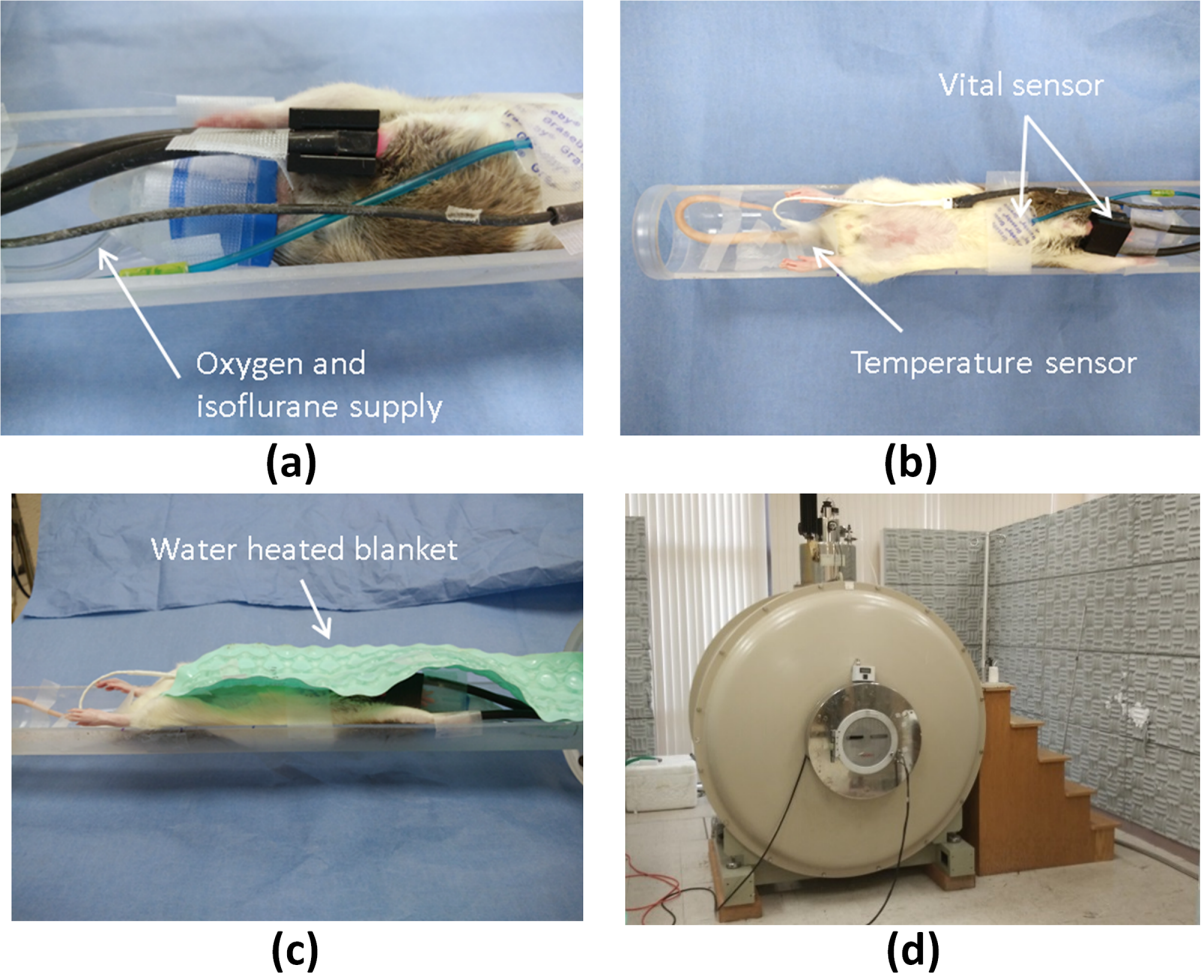
Experimental setup and Varian 4.7 T MR scanner. (a) The animal was anesthetized using isoflurane and oxygen mixture for MR measurements and radiation treatment. (b) The animal’s vitals (respiratory and pulse oximetry) and temperature were monitored. (c) The animal was covered by a water-heated blanket to keep the temperature stable during MR measurement. (d) Varian 4.7 T MR scanner used in the study.

MR imaging data were collected using a horizontal bore 4.7 T Varian MR scanner (Agilent Technologies, Santa Clara, CA, USA). A 2% isoflurane and oxygen mixture, with flow at 1.5 L/min, was used to induce and maintain general anesthesia in rats during MR measurements. The animal was placed on the custom-made acrylic cradle and covered with a temperature controlled water circulating vinyl blanket to maintain the body temperature at 37 °C. The cradle was placed inside a rat body Litzcage Coil (Doty Scientific, Columbia, SC), as illustrated in Fig 3. Anatomical T_2_-weighted MR sequence was used (Fast Spin Echo with echo train length of 8; TR = 7628 ms, Effective TE = 40 ms). Acquisition matrix was 128 × 128, and field of view was 70 mm × 70 mm x 1 mm giving an in-plane resolution of 0.547 mm × 0.574 mm after image reconstruction. Ninety axial images were obtained to cover the entire tumor volume with a slice thickness of 1 mm and no gaps or overlap between slices. Using 4 signal averages, the total scan time was 8 minutes 23 seconds.

#### CBCT image acquisition

XRad 225Cx (PXI, North Branford, CT, USA) on-board imaging was used to acquire CBCT data. The same concentration of isoflurane and oxygen mixture was used during CBCT acquisition and throughout the entire radiation treatment. Mimicking the MR scan position, the animal was positioned in the XRad 225Cx irradiator for a setup verification scan. The CBCT source-axis distance and source-detector distance were 30.5 cm and 64.5 cm, respectively. A 70 mm × 70 mm field of view (FOV) was used. The subject was scanned with a 40 kVp and 0.5 mA setting in approximately 30 seconds. The raw data were processed using the Feldkamp-Davis-Kress (FDK) reconstruction algorithm. The reconstructed image resolution was 0.2 mm × 0.2 mm and the slice thickness was also set to 0.2 mm. The final image matrix was 350 × 350 × 400 voxels.

#### MR image distortion correction

Combined MR and CBCT images were used to create a new MR-CBCT based IGRT procedure for preclinical small animal studies. Any artifacts and geometry distortions between the two imaging modalities were assessed before registering two types of images. The calibration phantom was used to characterize the geometry distortions, which were next validated by utilizing the geometry phantom images.

The calibration phantom, Fig 1(a), was first placed on the MR cradle, Fig 2(b), for a scan. The phantom was then placed on the compatible cradle, Fig 2(c), in XRad 225Cx irradiator. Based on the measured z-axis indicator inside the phantom, seventy MR axial images were aligned to the corresponding CBCT images. The affine transformation [28, 29] mapping MR coordinate system to CBCT coordinate system was computed on a slice-by-slice basis for all seventy axial planes using MATLAB (MathWorks, Natick, MA, USA).

The maximum imaging field of view of the preclinical MR scanner is small, approximately 70 mm in diameter and 70 mm in length. The geometry distortions were observed in many scanning protocols leading to erroneous subject dimensions or volume assessments. Primarily the geometry distortions originate from the inhomogeneous main magnetic field B_0_, and nonlinearities in the gradient magnetic field B_1_. An intrinsic object-related perturbation component is also present. Assuming that the distortion presented in the field would manifest itself as a continuous function without any local rapid changes in such small volume [30–32], a global transformation could properly describe the field distortion and generate the correction factors for scanning protocols. Affine transformation with 3D cubic b-spline interpolation was implemented in the study [33].

There are four components defining transformation—scale, translation, shear, and rotation. The computed axial slice-by-slice coordinate transformations in vertical and horizontal directions were interpolated along the z-axis using a 3D cubic b-spline basis function. A minimum of 4 control points are required for each coordinate axis interpolation, *i*.*e*., 4^3^ for a 3D space, therefore, there are 64 control points encompassing the phantom volume. This coordinate transformation was used to map the MR onto CBCT images accounting for the MR artifacts.

#### Animal study overview and workflow

The study was approved by the UT Southwestern Medical Center Institutional Animal Care and Use Committee (IACUC). A human prostate cancer cell line PC3-DABIP-luc was implanted into a male Copenhagen rat (12 weeks, 200–250 g, Charles River, Frederick, MD), as described in detail previously [34]. Briefly, the rat was anaesthetized with 2% isoflurane in oxygen. The abdomen was shaved and the remaining hair was removed with Nair^™^ razor (Church & Dwight Co., Inc., Ewing, NJ, USA). A suprapubic incision was used to expose the bladder and prostate. 5 × 10^5^ cells in serum-free medium for a total volume of 30 μL were injected directly into the prostate using a 0.36 mm outer diameter needle, *viz*., 28-gauge needle (Exelint International Co., Redondo Beach, CA, USA). A sterile cotton-tipped applicator was held over the injection site to avoid leakage. The fascia was closed with absorbable sutures (Henry Schein Animal Health, Dublin, OH, USA). The skin was closed with 9 mm AutoClips (MikRon Precision, Inc., Gardena, CA, USA) and Vetclose glue (Henry Schein Animal Health, Dublin, OH, USA).

Tumor growth was assessed weekly using a 4.7 T Varian MR scanner. On treatment day, MR of the animal subject was acquired first, immediately followed by the calibration phantom scan. The phantom scan was used to characterize the magnetic field distortions and to obtain the corresponding correction factors which were subsequently applied to that particular animal MR image set. In other words, the obtained correction factors are animal specific enabling the accurate targeting for radiotherapy.

For treatment, the animals were placed in the CBCT cradle in the position identical to the MR scan and a CBCT scan was acquired utilizing an XRad 225Cx irradiator. The co-registration between corrected MR (cMR) and CBCT images was done by XRad 225Cx built-in algorithm, namely Image Match. Co-registered images were further processed in MATLAB version R2017 to create fused cMR-CBCT data set, denoted as CBCT+ set, which could be imported into SmART-Plan. This procedure enables the structures to be contoured on a CBCT+ dataset, while the CBCT images are utilized for treatment planning and dose calculation.

#### MR-CBCT based SmART-Plan planning

**Fig 4:**
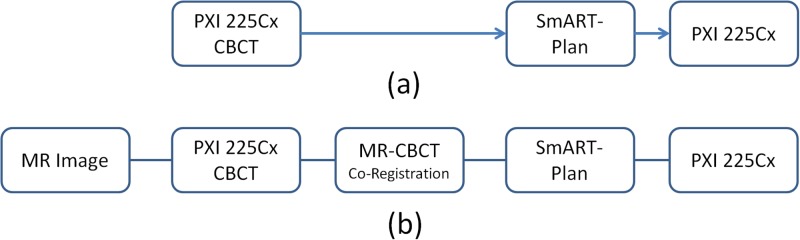
Imaging workflow. (a) A CBCT-based workflow and (b) In-house MR-CBCT-based workflow.

A CBCT based manufacturer workflow and a custom-designed MR-CBCT image guided workflow are illustrated in Fig 4. For the in-house procedure the corrected MR images (cMR) were imported into the XRad 225Cx image data base and the image co-registration between the acquired CBCT images and the imported cMR dataset was performed. The co-registered images, however, could not be readily sent to SmART-Plan and utilized for treatment planning, as intended by the manufacturer workflow scheme depicted in Fig 4(a). Additional data processing was performed in MATLAB to create readable co-registered MR-CBCT data for SmART-Plan. The major difference between the manufacturer (CBCT-based planning) and in-house (combined MR-CBCT-based planning) procedures is the implementation of a pre-treatment MR scan followed by the MR-CBCT co-registration, see Table 1 for abbreviated process summary.

**Table 1:** The steps for manufacturer and in-house procedures.

	Manufacturer Procedure	In-house Procedure
PXI XRad 225Cx	Acquire CBCT Export to SmART-Plan	Import distortion corrected MR (cMR) Acquire CBCT Perform “Image Match” between cMR and CBCT Record “Image Match” result Export CBCT to MATLAB
MR-CBCT Co-registration		Shift cMR based on “Image Match” result Fuse shifted cMR with CBCT to create CBCT+ (fused cMR-CBCT data set) Export both CBCT and CBCT+ to SmART-Plan
SmART-Plan	Contour Plan Export RT plan back to PXI for delivery	Contour on CBCT+ and save the structures Load CBCT and import the previously saved structures Plan Export RT plan to PXI for delivery

First, utilize the XRad 225Cx built-in registration algorithm called “Image Match” function. The function allows the operator to register the acquired CBCT with the pre-imported image data set, *e*.*g*., cMR, using rigid body translation and rotation. Once the “shift” is acquired the conventional procedure is to move the subject to the reference position by using motorized table. However, in the proposed MR-CBCT based planning procedure, the difference is not compensated by table correction. The co-registration result also represents the subject positioning difference between two imaging modalities. Therefore, instead of moving the table to bring the subject to cMR coordinate, the cMR images are translationally and rotationally shifted in three dimensions based on the “Image Match” results to co-register cMR in CBCT coordinate system. Once the “shift” is applied, the cMR and CBCT image are aligned in the same coordinate system.

Second, create CBCT+ image set, in other words the fused cMR-CBCT data set. Since SmART-Plan is only designed to work on CT images, a duplicated CBCT image DICOM framework was used to store CBCT+ data. The cMR intensity is normalized based on CBCT intensity to match the same dynamic range with CBCT during the fusing process. The two image data sets share the same coordinate system and dynamic range of signal intensity. Depending on study purpose, different ratios could be applied to emphasize structural features on either cMR or CBCT images.

Third, export both CBCT and CBCT+ datasets to SmART-Plan and create a treatment plan. Since both data sets are in the CT image format, they are now readable within SmART-Plan. The CBCT+ images are composed of cMR and original CBCT, the intensity in the image is used to assist structure identification, *i*.*e*., strictly for contouring, not dose computation. Once the contours are finalized, the contours are exported and applied to the original CBCT images to complete treatment planning and dose calculation.

## Results

### MR image distortion correction

**Fig 5:**
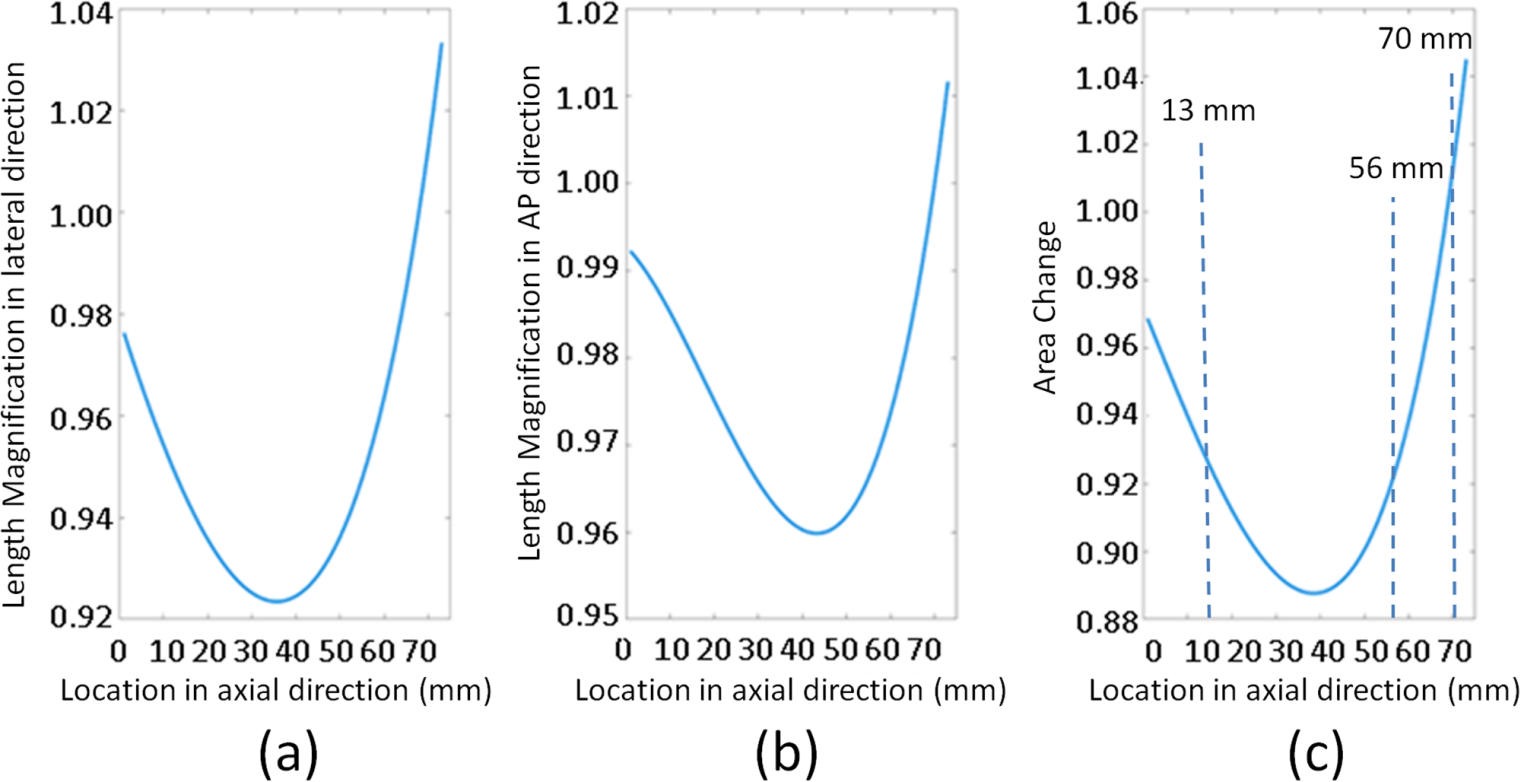
Length magnification in left-right (x-axis) and anterior-posterior (y-axis) directions and the area change along superior-inferior (z-axis) direction.

**Fig 6:**
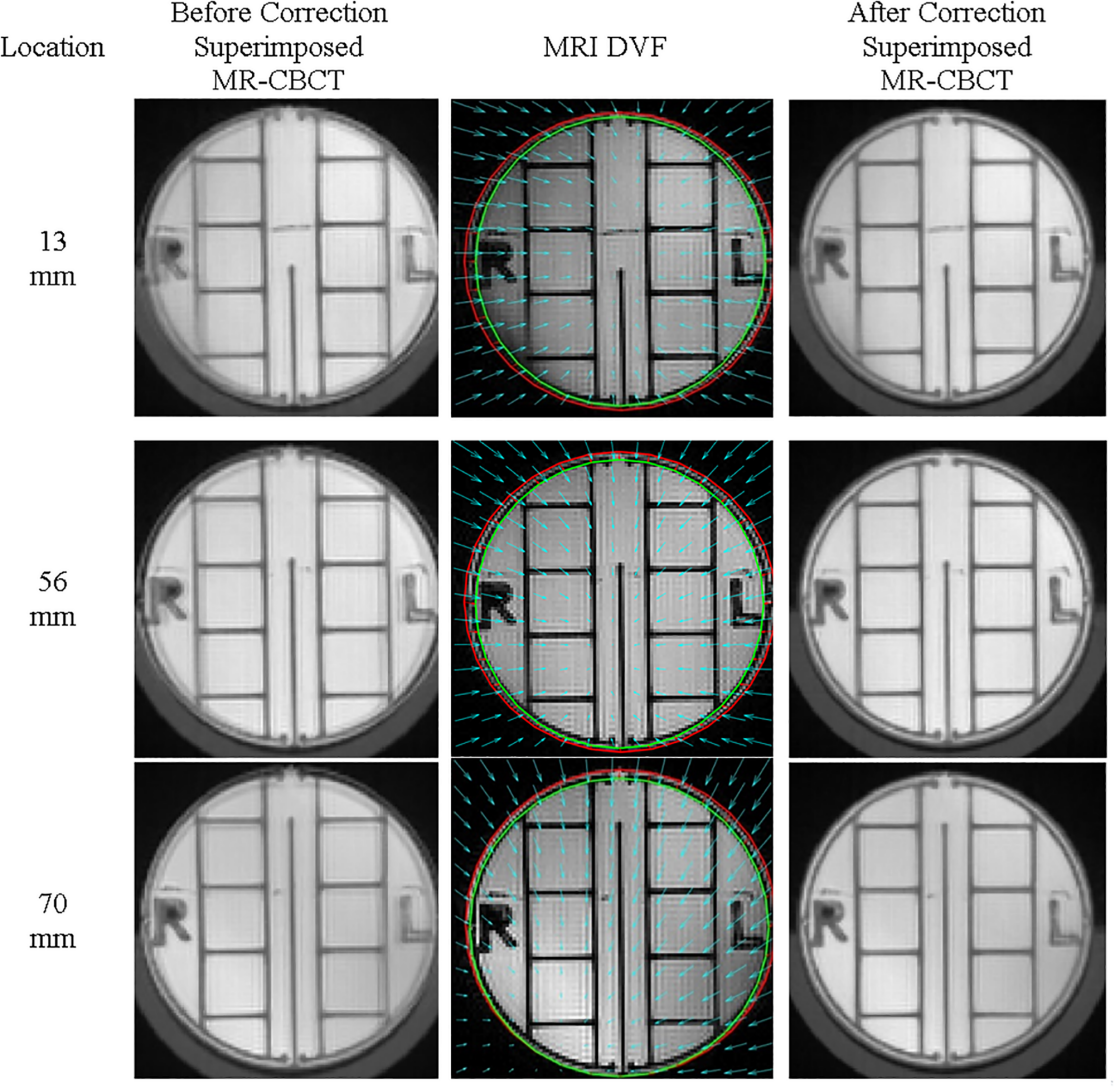
Characterization of magnetic field distortion at 13 mm, 56 mm and 70 mm. The cyan arrows represent the deformation vector fields (DVF). The red circles correspond to the original outline of the phantom in the MR images before correction, while the green circles correspond to the CBCT outline of the phantom.

The results of the affine transformation between MR and CBCT image sets for each of the seventy axial slices are shown in Fig 5. Fig 5(a) and 5(b) show scaling components of the affine transformation matrix along the z-axis, where the x and y axes correspond to the left-right (L-R) and anterior-posterior (A-P) directions, respectively. The product of length magnification in x and y directions of scaling component of the affine transformation matrix represents the corresponding area change. Fig 5(c) illustrates the change in area along the z-axis. Comparison between MR and CBCT images indicates the majority of MR geometric distortions arise from scale and rotation. The geometry distortions vary depending on the slice location along the longitudinal axis, *i*.*e*., z-axis of MR scanner. Thus, the structures in MR images could be either smaller or larger than the corresponding structures in CBCT images. The geometric distortion, Fig 5(c), varied between -5% and 11% throughout the scanning volume. Fig 6 illustrates the results before and after accounting for the length magnification along x and y axes. The calibration phantom images, in Fig 6, at locations 13 mm, 56 mm and 70 mm, correspond to dashed lines in Fig 5(c), respectively. The cyan arrows represent the deformation vector fields (DVF). The red circles correspond to the original outline of the phantom in the MR images before correction, while the green circles correspond to the CBCT outline of the phantom.

**Fig 7:**
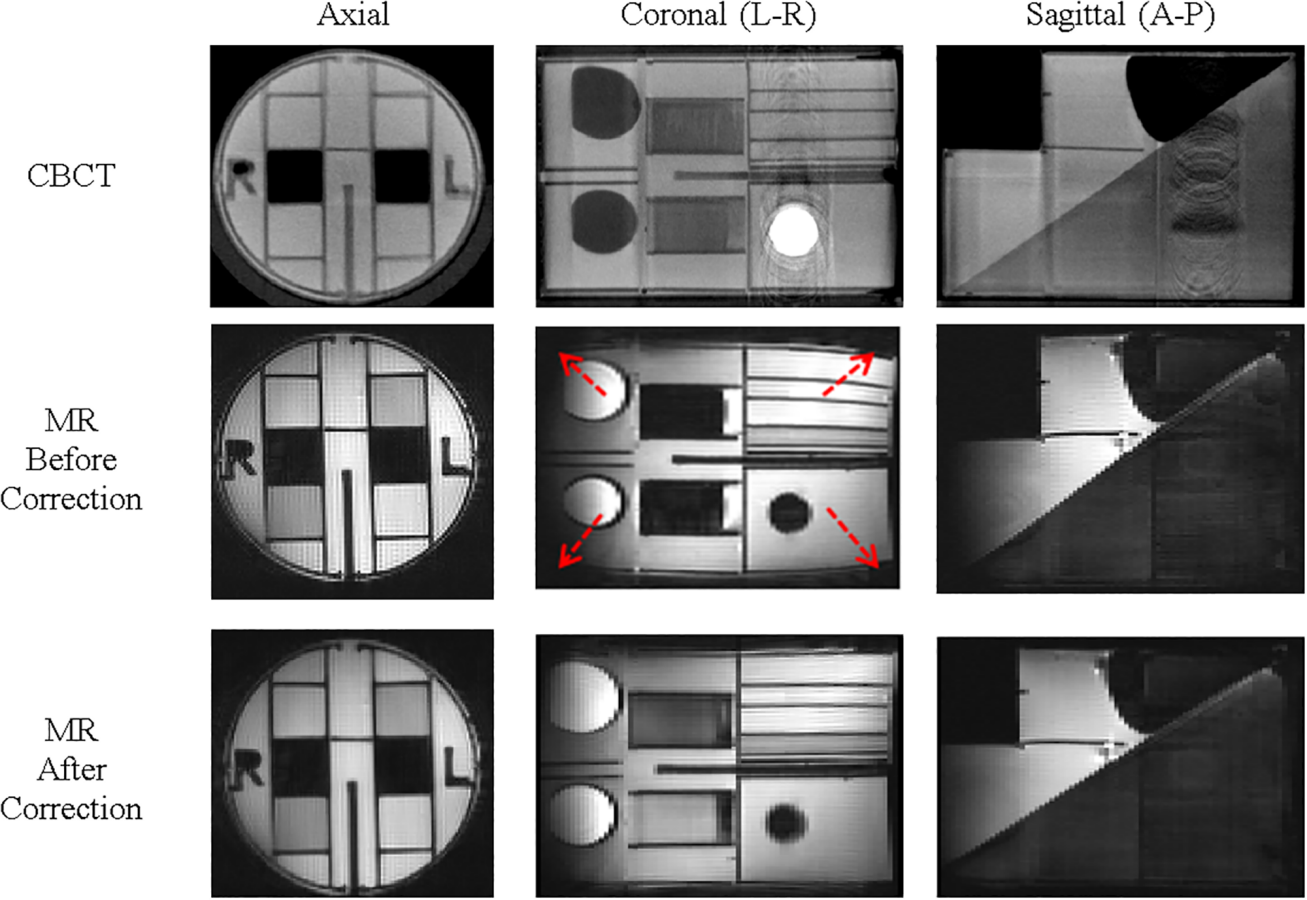
CBCT and MR geometry phantom images before and after applied correction. Arrows point to noticeable geometric distortion.

Fig 7 shows the reference CBCT and the corresponding MR images of the geometric phantom in axial, coronal, and sagittal planes before and after applying the geometric correction. While there is no evident distortion in the CBCT images, significant distortion is apparent in the MR before applying the corrections, specifically in the left-right and anterior-posterior directions of the coronal and sagittal planes, respectively. The geometry of coronal image is magnified in the middle of the imaging field, matching the calibration phantom results shown in Fig 5. After applying the correction, the geometry of the MR is restored.

**Fig 8:**
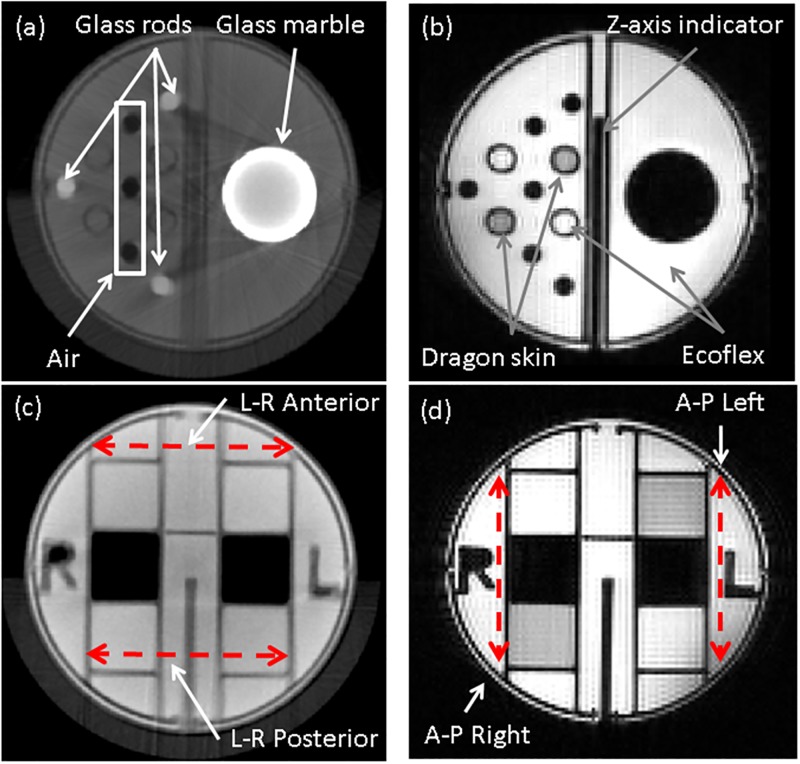
CBCT and the corresponding distortion corrected MR images. (a) CBCT and (b) MR of the contouring and contrast modules. (c) CBCT and (d) MR of the grid module.

Fig 8 shows CBCT images of the geometric phantom and the corresponding distortion corrected MR images. Fig 8(a) and 8(b) depict the contrast and contouring modules. Variations in the silicone compounds, Dragon Skin^®^ and Ecoflex^®^, result in a source of contrast identifiable via MR, but not CBCT images. The grid size measurements before and after geometry distortion corrections are listed in Table 2. Based on the geometry comparison results, the raw CBCT images were used as the reference images and considered the ground truth without any geometry correction in the animal studies. Two remarks are worth noting. First, the differences between CBCT and the blueprint design were within 0.1 to 0.4 mm, which is characterizing the fidelity of manufacturing 3D printing process. Second, while measured physical dimensions of scanned phantoms can be compared to the design using a caliper, equivalent measurements for image correction of animal subjects would be extremely challenging. Furthermore, there is no geometry ground truth for animals. Indeed CT/CBCT images are widely accepted as the reference standard for radiation therapy planning in the clinic. Therefore, the CBCT images were used as the ground truth for MR image correction and as the reference for co-registration.

**Table 2:** Grid size measurements before and after correction.

		Blueprint [mm]	CBCT [mm]	Uncorrected MR [mm]	Difference [%]	Corrected MR [mm]	Difference [%]
L-R	Anterior	30.00	30.23	32.64	8.0%	30.18	-0.2%
	Posterior	30.00	30.42	32.81	7.9%	30.30	0.4%
A-P	Right	32.00	31.94	32.61	2.1%	31.92	-0.1%
	Left	32.00	31.83	32.53	2.2%	31.84	0.0%

### Image co-registration and distortion correction

**Fig 9:**
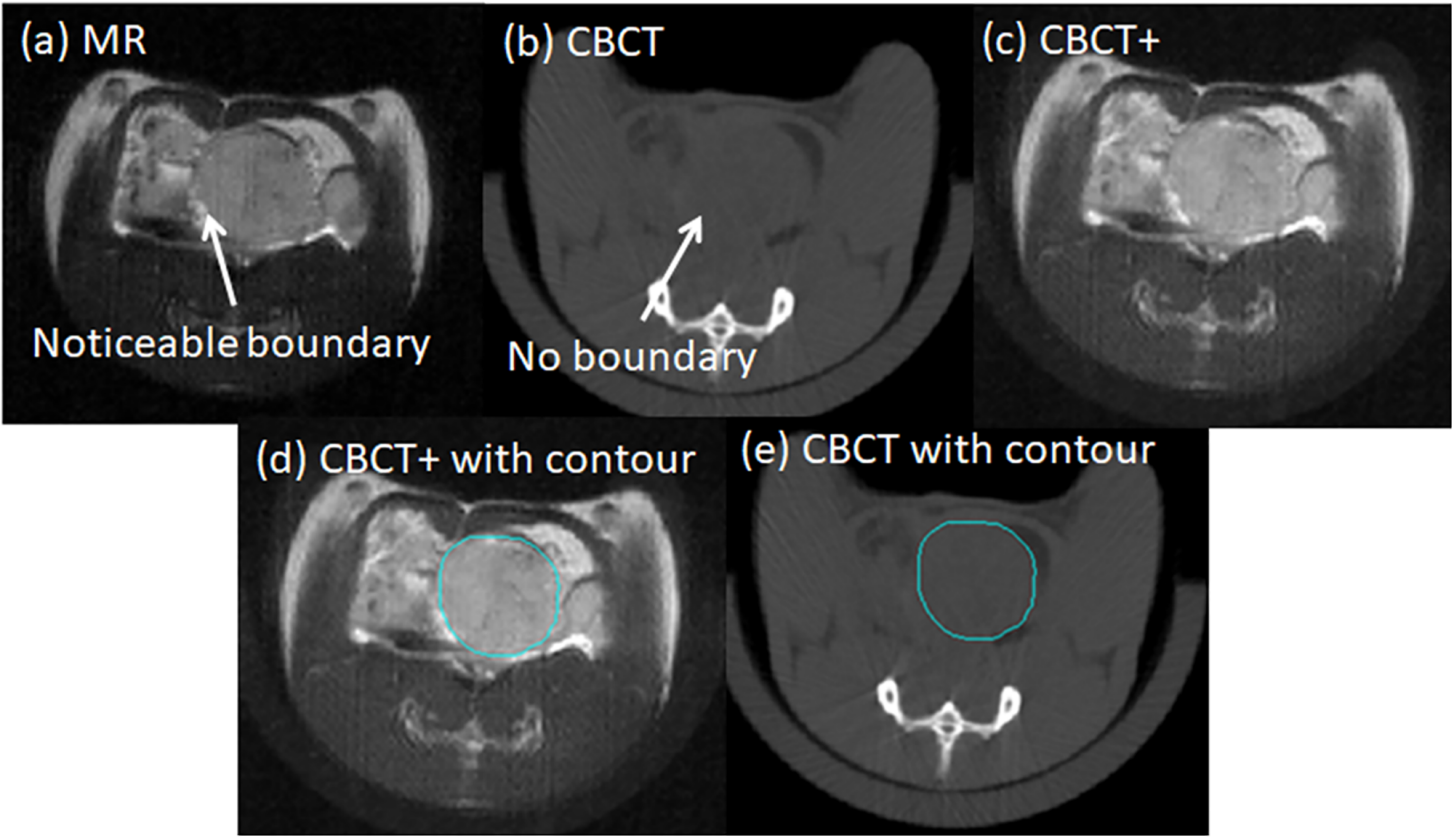
Planning workflow. (a) and (b) are the MR and CBCT data sets, respectively, used for co-registration. (c) MR structural features superimposed on the corresponding CBCT image labeled the CBCT+ dataset. (d) CBCT+ with the manually delineated planning target volume (PTV) contour. (e) CBCT image with PTV contour for final dose calculation and statistical analysis.

The tumor boundary was readily identified in the MR image, Fig 9(a), but difficult to define based on the original CBCT image alone, Fig 9(b). The CBCT+ image in Fig 9(c) shows the noticeable tumor boundary to be used for contouring. The tumor contour is drawn on the CBCT+ image data set, Fig 9(d), and then exported back to the original CBCT image data set for dose calculation, Fig 9(e).

**Fig 10:**
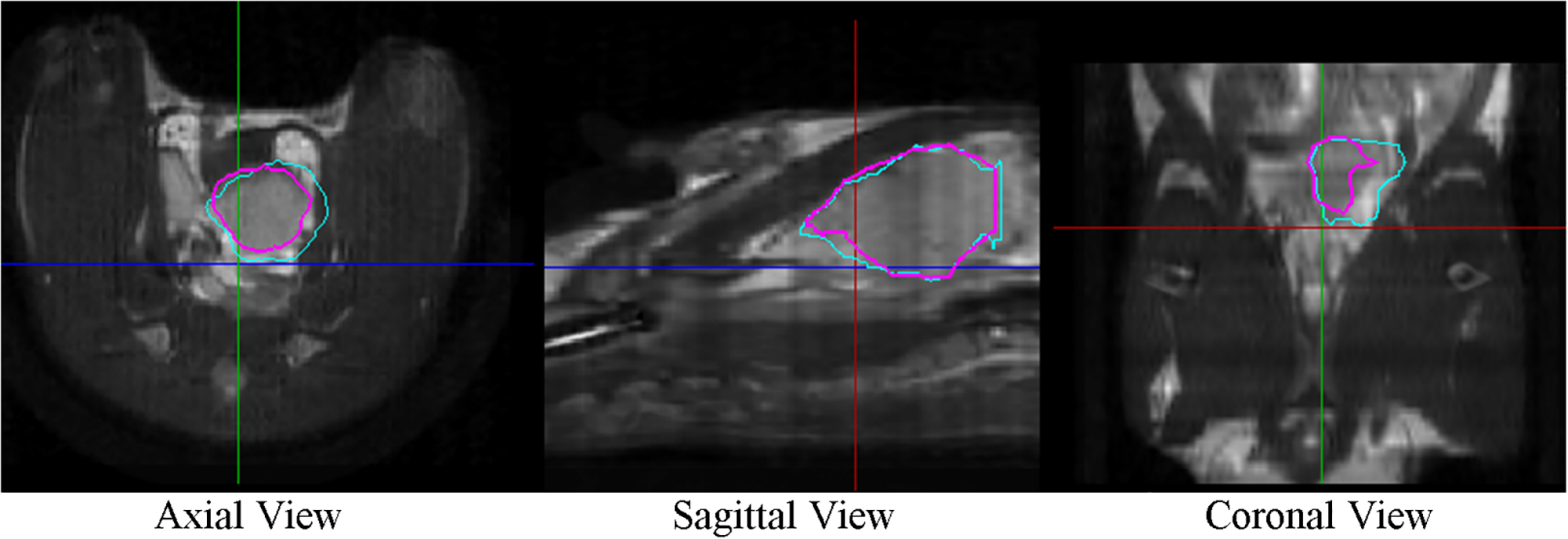
PTV contours displayed on the distortion corrected fused MR-CBCT image. Cyan represents PTV before correction, while pink depicts PTV after correction.

To quantify the impact from the static magnetic field distortion, three blind tests were performed. Three planners were asked to contour the PTV on the fused MR-CBCT images with and without distortion correction applied. Fig 10 illustrates one of the three results demonstrating the inconsistent PTV contours on the CBCT+ images. The cyan structure was contoured on the fused MR-CBCT images without distortion correction then imported to the CBCT+ images, whereas the pink structure shows PTV derived from corrected images. The relative difference in the estimated PTV volumes with and without magnetic field distortion corrections are shown in Table 3. The PTV volumes were smaller for all three planners by 16% to 22% after applying the distortion correction.

**Table 3:** Tumor delineation blind test.

	PTV volume (cc)		
Planner	Before MR Correction	After MR Correction	Percent Change
1	1.66	1.30	-21.7%
2	1.87	1.52	-18.7%
3	2.17	1.82	-16.1%

### Treatment plan

Conformal arc therapy, using 225 kVp photons, was planned with 9 partial arcs in 40 degree increments to avoid possible hardware issues observed when delivering a full 360-degree arc at once. A 15 mm circular cone was used for treatment planning and delivery. Without a clear PTV boundary, it is difficult to select the proper cone for the treatment. If a cone is too small, the tumor may not be properly treated, while a cone too large may result in higher toxicity.

**Fig 11:**
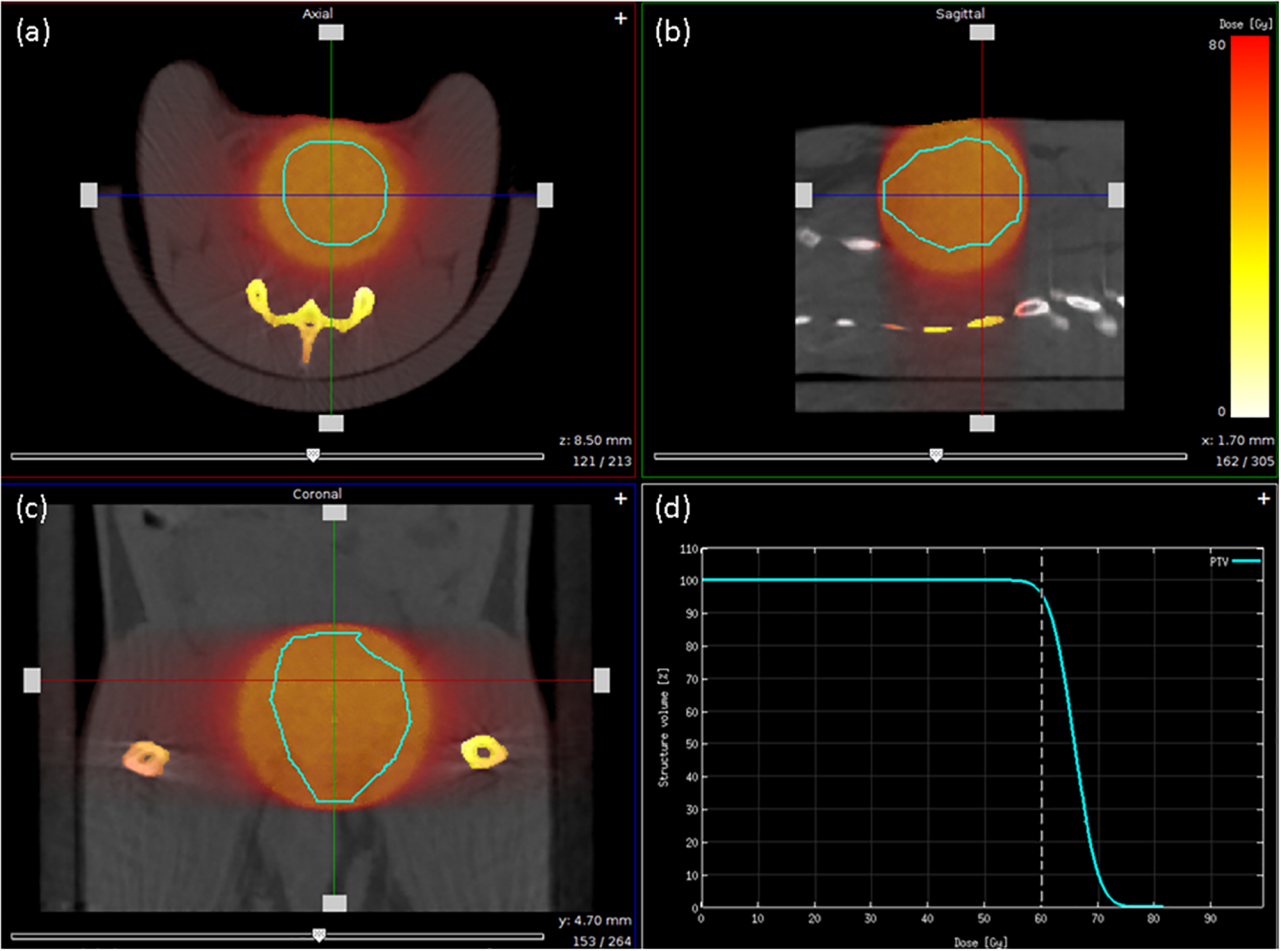
Dose distribution and dose volume histogram for Rx of 60 Gy to 95% of PTV. (a), (b), and (c) correspond to axial, sagittal, and coronal planes. A 15 mm circular cone was used for treatment planning and delivery. (d) DVH representation of PTV coverage.

Fig 11(a), 11(b) and 11(c) illustrate the resulting dose distribution in axial, sagittal, and coronal views, respectively. Fig 11(d) shows the PTV dose volume histogram (DVH). In this case, the 95% of the PTV volume was covered by 60 Gy.

## Discussion and conclusion

The proposed procedure is an add-on function that enables use of dual modality MR-CBCT image guidance for XRAD 225Cx SmART-Plan in a preclinical setting. This novel protocol is wide-ranging and not limited to the orthotopic prostate tumors presented here. The current design requires MR and CBCT scanner dedicated cradles, introducing setup uncertainties during the transfer of rats between two cradles. An interchangeable cradle is under design. For future studies, the animal would stay in the same cradle from imaging through the treatment to minimize setup errors.

To evaluate both imaging systems, two MR-CBCT compatible imaging phantoms were designed and custom-made for commissioning and end-to-end testing. A calibration phantom was used to characterize the geometry distortions originating from the main B_0_ and gradient B_1_ magnetic fields, *i*.*e*., it is used to compute the coordinate transformation between the MR and CBCT data, while a geometric phantom was used to verify the findings. The PTV blind test in this study revealed that the PTV volume was on average 19% smaller when contoured on the corrected cMR-CBCT images relative to raw images without distortion correction. Consequently, a 15 mm circular cone was suitable for treatment planning and delivery, whereas a larger 20 mm circular cone would have been selected for the uncorrected images causing increased toxicity to the normal tissue. In general, misleading target volumes and organs at risk (OARs) delineation could adversely impact the reproducibility of the reported results and the study conclusions.

Unfortunately, there is no universal geometry distortion correction applicable to an arbitrary animal MR scan. The geometry distortion corrections encompass not only the inhomogeneous main magnetic field B_0_ but also the imperfect gradient field B_1_ and the custom preclinical MR scanner shim settings. The underlying limitations of the method are the coupled B_0_ and B_1_ inhomogeneity and the susceptibility artifacts stemming from differences in phantom structures relative to the actual animal composition. The susceptibility errors due to animal tattoos or surgical clips could be present in the corrected images.

Combined MR-CBCT data offer many potential benefits compared to CBCT images alone. There is extensive evidence that hypoxia can influence radiation response. Recent work has shown that MR parameters such as blood oxygenation level dependent (BOLD), tissue oxygenation level dependent (TOLD), and dynamic contrast enhancement (DCE) [35–39] may be correlated with radiation response, potentially providing prognostic imaging biomarkers. More invasive imaging procedures exploiting exogenous reporter agents have shown strong correlations between radiation response and hypoxia [40–43]. As part of the treatment plan, one may ultimately envisage dose painting as local radiation boost to hypoxic regions [44, 45]. Although MR image-guided radiotherapy is regularly applied in the clinic, both hardware and software have limited application in preclinical small animal research. Considering that small animal research focuses on much smaller subjects than humans, a slight over-dose or under-dose could make a significant impact on outcome. Over-dosing could result from overestimating the target volume, which results in radiation injury to the normal tissue. One the other hand, under-dosing could result in poorer tumor control outcomes. Both scenarios dramatically increase the uncertainty on research results. Therefore, an MR image-guided radiotherapy for preclinical small animal research is necessary, especially for soft tissue regions such as lung, kidney, liver, or prostate.

## Supporting information

S1 File Simulation_cradle.stl. (STL)

S2 File Calbiration_phantom_left_grid.stl. (STL)

S3 File Calibration_phantom_right_grid.stl. (STL)

S4 File Calibration_phantom_shell.stl. (STL)

S5 File Geometry_phantom_hidden_target_module.stl. (STL)

S6 File Geometry_phantom_left_grid_module.stl. (STL)

S7 File Geometry_phantom_right_grid_module.stl. (STL)

S8 File Geometry_phantom_shell.stl. (STL)

S9 File Geometry_phantom_contrast_module.stl. (STL)

S10 File Water_chamber.stl. (STL)
